# Ginsenoside Rg1 Prevents Doxorubicin-Induced Cardiotoxicity through the Inhibition of Autophagy and Endoplasmic Reticulum Stress in Mice

**DOI:** 10.3390/ijms19113658

**Published:** 2018-11-20

**Authors:** Zhi-Meng Xu, Cheng-Bin Li, Qing-Ling Liu, Ping Li, Hua Yang

**Affiliations:** State Key Laboratory of Natural Medicines and School of Traditional Chinese Pharmacy, China Pharmaceutical University, Nanjing 210009, China; jorjames@126.com (Z.-M.X.); licb1993@126.com (C.-B.L.); xl9696xl@163.com (Q.-L.L.)

**Keywords:** ginsenoside Rg1, doxorubicin, cardiotoxicity, autophagy, endoplasmic reticulum stress

## Abstract

Ginsenoside Rg1, a saponin that is a primary component of ginseng, has been demonstrated to protect hearts from diverse cardiovascular diseases with regulating multiple cellular signal pathways. In the present study, we investigated the protective role of ginsenoside Rg1 on doxorubicin-induced cardiotoxicity and its effects on endoplasmic reticulum stress and autophagy. After pre-treatment with ginsenoside Rg1 (50 mg/kg i.g.) for 7 days, male C57BL/6J mice were intraperitoneally injected with a single dose of doxorubicin (6 mg/kg) every 3 days for four injections. Echocardiographic and pathological findings showed that ginsenoside Rg1 could significantly reduce the cardiotoxicity induced by doxorubicin. Ginsenoside Rg1 significantly inhibited doxorubicin-induced formation of autophagosome. At the same time, ginsenoside Rg1 decreased the doxorubicin-induced cardiac microtubule-associated protein-light chain 3 and autophagy related 5 expression. Ginsenoside Rg1 can reduce endoplasmic reticulum dilation caused by doxorubicin. Compared with the doxorubicin group, the expression of cleaved activating transcription factor 6 and inositol-requiring enzyme 1 decreased in group ginsenoside Rg1. Treatment with ginsenoside Rg1 reduces the expression of TIF1 and increases the expression of glucose-regulated protein 78. In the ginsenoside Rg1 group, the expression of p-P70S6K, c-Jun N-terminal kinases 1 and Beclin1 declined. These results indicate that ginsenoside Rg1 may improve doxorubicin-induced cardiac dysfunction by inhibiting endoplasmic reticulum stress and autophagy.

## 1. Introduction

Doxorubicin is a widely used and highly successful anticancer chemotherapeutic drugs agent. Unfortunately, its clinical use is critically limited by its cumulative cardiotoxicity [1]. It is well established that cancer patients undergoing doxorubicin treatment are susceptible to cardiac anomalies, including hypotension, tachycardia, arrhythmia, and transient depression of left ventricular function [2]. Therefore, a major challenge in managing cancer patients treated with doxorubicin is to minimize doxorubicin’s cardiotoxic effects without compromising its antitumor properties.

Autophagy is a conserved process aimed at maintaining cell and tissue homeostasis by degrading and recycling macromolecules, organelles and nutrients. Previous studies have shown that dysregulated autophagy is associated with a variety of heart diseases including ischemia/reperfusion injury, cardiac hypertrophy, cardiac aging, and heart failure [3]. However, the accumulation of oxidized and damaged macromolecules and organelles induced by doxorubicin can lead to autophagy in cardiac myocytes [4]. It has been reported that the suppression of autophagy could attenuate doxorubicin-induced cardiotoxicity [5,6,7]. In addition, pharmacologic inhibition of post- doxorubicin (DOX) autophagy by 3-methyladenine protected cardiac cells from DOX toxicity [4]. Thus, decrease of autophagy could be a potential strategy for the treatment of DOX-induced cardiomyopathy.

The endoplasmic reticulum (ER) is a membranous organelle supporting many processes required by almost every mammalian cell, including cardiomyocytes. Various factors that interfere with ER functions (protein folding, lipid and sterol synthesis, and Ca^2+^ homeostasis) lead to ER stress via the activation of complex cytoplasmic and nuclear signaling pathways collectively termed the unfolded protein response (UPR) [8]. The UPR initially activates three major signal transducers, viz. type I transmembrane ER-resident protein kinase (PERK), activating transcription factor 6 (ATF6), and Inositol-requiring enzyme 1 (IRE1) [9]. Recent study has revealed that the perturbation of UPR is implicated in the pathophysiology of doxorubicin-induced cardiotoxicity [10,11]. DOX inactivates glucose-regulated protein 78 (GRP78) leading to the activation of ATF6 and IRE1, then cause cardiomyocyte apoptosis. Moreover, the administration of the chemical ER chaperone 4-phenylbutyrate alleviated cardiac apoptosis and dysfunction induced by doxorubicin. This suggests that inhibition of endoplasmic reticulum stress is a feasible way to improve DOX-induced cardiotoxicity.

Ginsenoside Rg1, one of the main components of ginseng, has been proved to counteract a variety of heart diseases [12,13,14]. In addition, its myocardial protective effect is related to the regulation of autophagy [15]. In addition, ginsenoside Rg1 can inhibit endoplasmic reticulum stress in Alzheimer′s disease and diabetes rat heart [16,17]. Latest research shows that oral administration of Rg1 decreased the cardiac cell apoptosis caused by doxorubicin and improved the cardiac function [18]. However, whether ginsenosides can improve DOX-induced cardiotoxicity by inhibiting autophagy and endoplasmic reticulum stress remains unknown. In a mice model of DOX-induced cardiomyopathy, we tested the hypothesis that ginsenoside Rg1 could alleviate DOX-induced cardiomyopathy by modulating autophagy and endoplasmic reticulum stress.

## 2. Results

### 2.1. Ginsenoside Rg1 Improved Cardiac Function in Mice Treated with Doxorubicin

**Figure 1:**
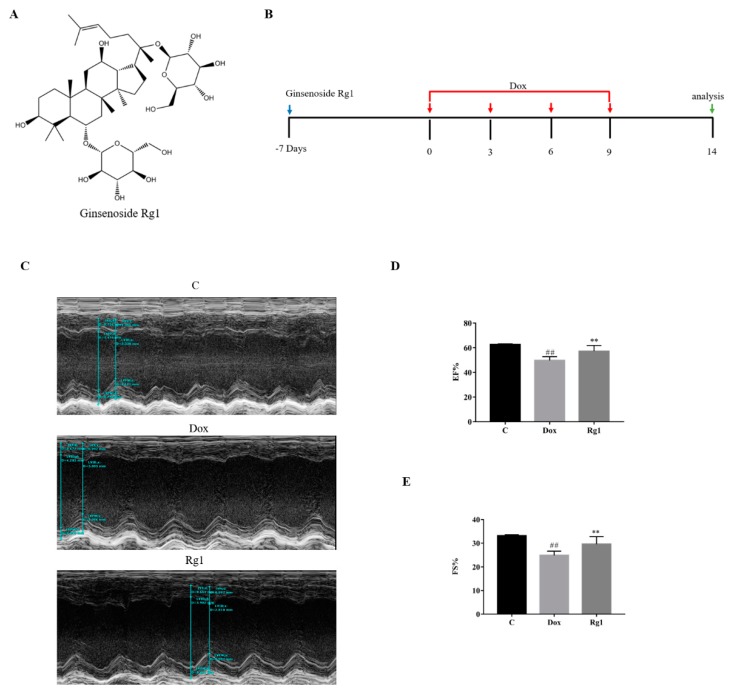
(**A**) Chemical structures of ginsenoside Rg1. (**B**) Schedule of the experiments. The mice received four doses of doxorubicin (6 mg/kg) or PBS every 3 d, as indicated. (**C**) Representative echocardiography images of left ventricle, (**D**) Echocardiographic measurement of EF (ejection fraction), (**E**) Echocardiographic measurement of fractional shortening (FS). ^##^ *p* < 0.01 vs C group, ** *p* < 0.01 vs. DOX group.

As shown in Figure 1, echocardiography images and quantitative analysis of the doxorubicin group demonstrated a significant impairment of left ventricular function expressed as ejection fraction (EF) and fractional shortening (FS) compared with the control group. Cotreatment with ginsenoside Rg1 with doxorubicin resulted in a remarkable improvement in EF and FS.

### 2.2. Ginsenoside Rg1 Alleviated Myocardial Pathological Changes and Cardiac Fibrosis in Mice Treated with Doxorubicin

**Figure 2:**
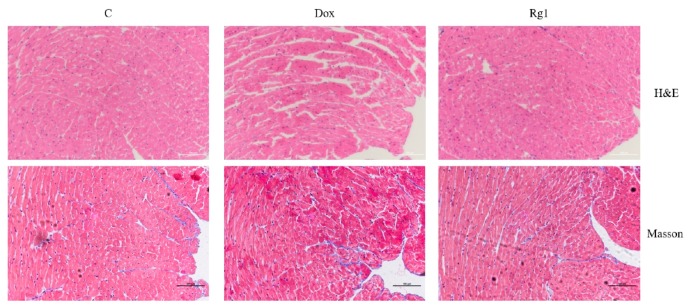
Protective effects of ginsenoside Rg1 on cardiac morphology and fibrosis in doxorubicin-induced cardiomyopathy. The upper panel represents images with H&E staining; the lower panel indicates cardiac fibrosis with Masson′s Trichrome staining. Scale bar: 100 μm.

Hematoxylin-eosin (HE) results showed that DOX-treated hearts displayed myofibrillar degeneration and disruption when compared with normal control hearts (Figure 2). Ginsenoside Rg1 treatment can significantly improve the defects induced by DOX. In addition, Masson′s Trichrome staining showed that the cardiac fibrosis in DOX group mice increased compared with the normal control group. However, the myocardial fibrosis in the ginsenoside Rg1 group was lower than the DOX group.

### 2.3. Ginsenoside Rg1 Inhibits Cardiac Autophagy in Mice Treated with Doxorubicin

**Figure 3:**
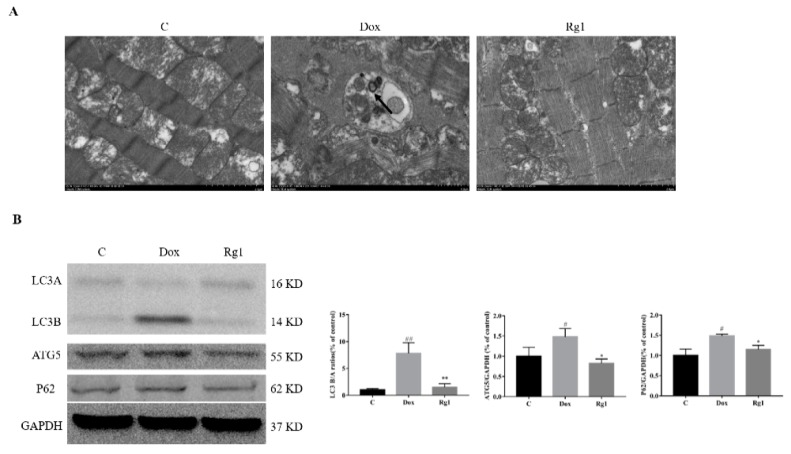
Rg1 inhibits autophagy in the hearts of mice. (**A**) Electron microscope images of autophagosome (indicated by arrow, 50,000×) from three groups. (**B**) Expression of LC3, ATG5 and P62 protein in mouse heart. GAPDH was used as the loading control. ^#^ *p* < 0.05, ^##^ *p* < 0.01 vs C group, * *p* < 0.05, ** *p* < 0.01 vs DOX group.

Electron microscopic images of the DOX-treated hearts showed the formation of autophagosome. This was not obvious in both the normal control group and the Rg1 treatment group (Figure 3A). Microtubule-associated light chain 3 (LC3) was always utilized as an index of autophagy function. Compared with the normal group, an increase of conversion of LC3A to LC3B was observed in the DOX group. Cotreatment with Rg1 can suppress this conversion caused by DOX ((Figure 3B). Moreover, DOX can cause an increase in the expressions of autophagy related 5 (ATG5) and sequestosome 1 (P62) in mice heart. Also, Rg1 reduces the increase in the expressions of ATG5 and P62 caused by DOX (Figure 3B).

### 2.4. Ginsenoside Rg1 Improved DOX-Induced Endoplasmic Reticulum Stress in Mice Heart

**Figure 4:**
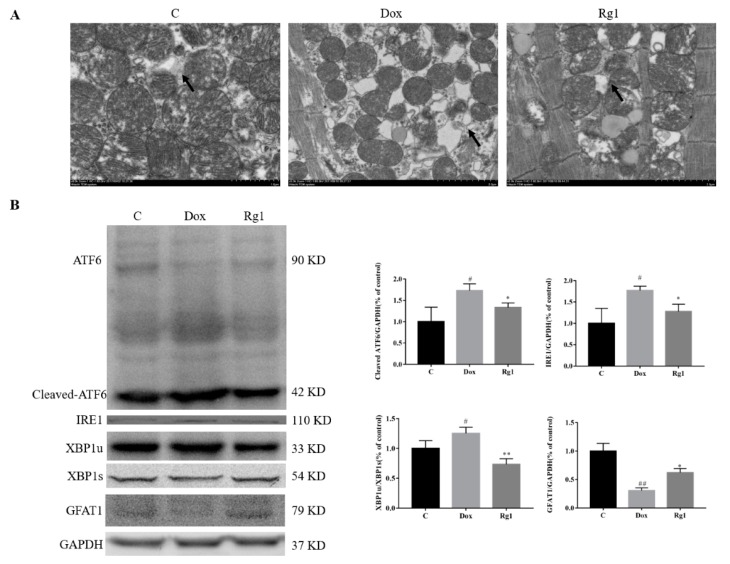
The anti-endoplasmic reticulum stress effects of Rg1 in mice heart. (**A**) Electron microscope images of ER (indicated by arrow, 50,000×) from three groups. (**B**) Expression of ATF6, Cleaved ATF6, IRE1, XBP1s, XBP1u and GFAT1 protein in mouse heart. GAPDH was used as the loading control. ^#^ *p* < 0.05, ^##^ *p* < 0.01 vs. C group, * *p* < 0.05, ** *p* < 0.01 vs DOX group.

Consistent with previous reports [10], we confirmed that doxorubicin resulted in marked ER dilation in mouse hearts. Rg1 administration can reduce the ER dilation (Figure 4A). It was found that DOX could increase the contents of cleaved ATF6 and IRE1 by protein expression detection. In the Rg1 group, the expressions of cleaved ATF6 and IRE1 were lower than that in the DOX group (Figure 4B). In addition, Rg1 can prevent the decrease of expressions of spliced X-box binding protein 1 (XBP1s) and glutamine fructose-6-phosphate amidotransferase (GFAT1) caused by DOX (Figure 4B).

### 2.5. The Mechanisms of Rg1 to Improve Endoplasmic Reticulum Stress 

**Figure 5:**
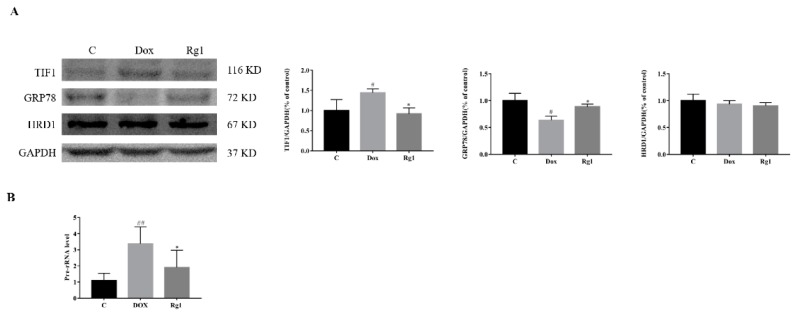
The mechanisms of Rg1 to improve endoplasmic reticulum stress. (**A**) Expression of TIF1, GRP78 and HRD1 protein in mouse heart. (**B**) Expression of Pre-rRNA level in mouse heart, GAPDH was used as the loading control. ^#^ *p* < 0.05, ^##^ *p* < 0.01 vs C group, * *p* < 0.05 vs DOX group.

It is generally known that the accumulation of misfolded proteins in the ER is alleviated by increasing expression of ER chaperones, arresting mRNA translation, and stimulating a process named ER-assisted degradation (ERAD) [19]. Compared with the normal group, DOX increased the expression of transcriptional intermediary factor (TIF1, mRNA translation) and reduced the expression of GRP78 (ER chaperone). In contrast, cotreatment with ginsenoside Rg1 can reduce the expression of TIF1A and increase the expression of GRP78 (Figure 5A). Furthermore, we found that Rg1 could inhibit the DOX-induced increase in the level of Pre-RNA (Figure 5B). However, there was no significant difference in expression of hydroxymethyl glutaryl-coenzyme A reductase degradation protein 1 HRD1, ERAD) between all groups (Figure 5A).

### 2.6. Effects of Rg1 on the Autophagic Pathway Activated by Endoplasmic Reticulum Stress in Mice Hearts 

**Figure 6:**
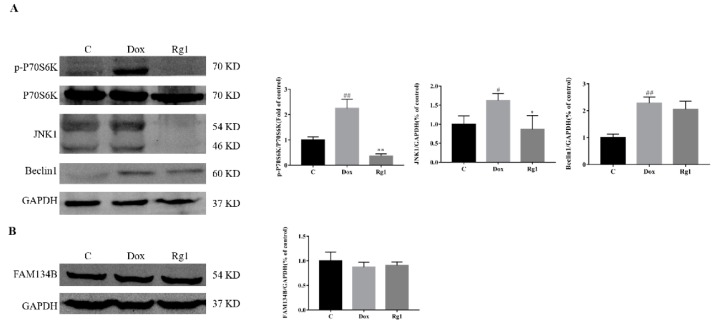
Effect of Rg1 on the autophagic pathway activated by endoplasmic reticulum stress in mice heart. (**A**) Expression of p-P70S6K, P70S6K, JNK1 and Beclin1 protein in mouse heart. (**B**) Expression of FAM134B protein in mouse heart, GAPDH was used as the loading control. ^#^ *p* < 0.05, ^##^ *p* < 0.01 vs C group, * *p* < 0.05, ** *p* < 0.01 vs. DOX group.

As reviewed by Rashid et al., the endoplasmic reticulum signaling regulate autophagy mainly by mTOR and Beclin1 [20]. Compared with the normal group, the expression of phosphorylated pibosomal protein S6 kinase beta-1 (p-P70S6K), c-Jun N-terminal kinases (JNK1) and Beclin1 increased significantly. Administration of Rg1 significantly reversed the expression alterations of these proteins (Figure 6A). However, there was no significant difference in expression of family with sequence similarity of 134 member B (FAM134B) between all groups (Figure 6B).

## 3. Discussion

Macroautophagy (commonly referred to as autophagy) is a conserved process from yeast to mammals for maintain cell and tissue homeostasis under normal as well as stress conditions, including nutrient starvation, changes in metabolism, and energy and oxygen status [21]. The role of autophagy in heart failure has been widely studied [22,23,24]. For example, Gao et al. found reverse upregulation of autophagy can reduce cardiomyocyte death in pressure-overload induced heart failure [23]. However, studies addressing the alterations in autophagic flux affect the cardiomyocyte response to doxorubicin have shown conflicting results, with doxorubicin-induced autophagy reported to be either increased or decreased [4]. Which indicate that the effects of autophagy on DOX-induced cardiotoxicity are very complicated. Recently, Rg1 has been reported to be able to improve DOX-induced decline in cardiac function in mice [18]. In addition, Rg1 can inhibit autophagy in H9c2 cardiomyocytes exposed to hypoxia/reoxygenation [15]. Therefore, the effect of Rg1 on autophagy in the heart of mice treated with DOX was observed. Compared with the normal control group, doxorubicin obviously induced autophagosome formation. However, the autophagosome is not apparent in the Rg1 group. LC3A, an immature isoform, is lipidated and processed to the mature LC3B through conjugation with phosphatidylethanolamine (PE), which further binds to the autophagosomal membrane and aids with autophagosomal elongation and maturation. DOX treatment has been reported to increase LC3B [4]. In our experiment, doxorubicin increased the ratio of LC3B/LC3A. In the Rg1 group, the ratio of LC3B/LC3A was lower than that in the DOX group. P62, a ubiquitin-binding cargo receptor, is targeted to the LC3-autophagosomal membrane interface through an LC3 binding domain and further facilitates autophagosomal maturation. In our experiments, Rg1 can reduce the expression of P62. Furthermore, Rg1 administration can also reduce the expression of ATG5, an E3 ubiubiquitin-like ligase necessary for autophagy. These indicated that Rg1 inhibits the autophagy induced by DOX in mice heart.

It is increasingly clear that endoplasmic reticulum stress participated in cardiac dysfunction induced by DOX [10,11]. As Rg1 can inhibit endoplasmic reticulum stress in rat heart [17], we assume that Rg1 can improve endoplasmic reticulum stress induced by DOX. We found that Rg1 treatment attenuated ER dilation induced by DOX. DOX-induced endoplasmic reticulum stress in cardiac myocytes mainly by affecting ATF6 cleavage and IRE1 [10]. However, Rg1 administration can reduce the increase in these two proteins content caused by DOX. Upon sensing the accumulation of unfolded proteins, IRE1α cleaves a cryptic exon of 26 bp from the downstream target gene X-box binding protein 1 (Xbp1) [25,26]. In the detection of XBP1s, we found that the expression of XBP1s in DOX group was reduced. In addition, Rg1 can increase the expression of XBP1s. GFAT1, the rate-limiting enzyme of the hexosamine biosynthetic pathway, is a direct target of Xbp1s in heart, and contributes to Xbp1s-mediated cardioprotection against I/R [27]. In our experiment, the expression of GFAT1 in the heart of the DOX group was significantly decreased. Also, Rg1 increased the expression of GFAT1. These results suggest that Rg1 can inhibit the overactivation of endoplasmic reticulum stress caused by DOX.

Once activated, the ER stress response retards protein translation, increases ER chaperone production, and enhances ER-associated protein degradation (ERAD), which together serve to increase the capacity of the ER and resolve the stress [8,9]. To find the way for Rg1 to improve endoplasmic reticulum stress in our study, we examined several key molecules which may be involved in it. Recently, transcription factor TIF1 has been associated with endoplasmic reticulum stress in heart. Inhibition of its activity led to attenuated endoplasmic reticulum (ER) stress and cell death [28]. In our experiment, DOX caused a significant increase in the TIF1 of mice hearts. Cotreatment with Rg1 reduce the increase in TIF1 caused by DOX. Simultaneously, we found that the level of Pre-rRNA in the Rg1 group was significantly lower than that in the DOX group. These results suggest that Rg1 may alleviate endoplasmic reticulum stress by inhibiting protein synthesis. GRP78 plays a pivotal role in the adaptive responses to ER stress by promoting protein folding [29]. We found that the expression of GRP 78 in the heart of the DOX group was significantly decreased when compared with the normal group. However, administration of Rg1 can increase the expression of GRP78. This suggests that the increase in protein folding may be one of the reasons for Rg1′s resistance to endoplasmic reticulum stress. ERAD is a quality control process for removing terminally misfolded proteins from the ER by the cytosolic ubiquitin-proteasome system [30]. Previous studies have shown HRD1 to play a key role in ERAD-mediated degradation of a wide spectrum of misfolded proteins [31]. In addition, HRD1 was the only ER transmembrane E3 ubiquitin ligase that was induced by ATF6 in heart [32]. However, we did not detect significant changes in HRD1 between each group. Therefore, the effect of Rg1 on the mitigate endoplasmic reticulum stress induced by DOX may be related to the regulation of protein synthesis and folding process. As we found that Rg1 had no significant effect on DOX-treated cardiomyocytes in vitro. The cause of Rg1 to improve endoplasmic reticulum stress induced by DOX needs further study.

Previous studies have shown that ER stress is closely related to the activation of autophagy [20]. There are two main types of autophagy induced by endoplasmic reticulum stress, ER stress-mediated autophagy and ER-phagy [33]. The signaling pathways of UPR are necessary for the activation of ER stress-mediated autophagy, while the receptor-mediated selective ER-phagy degrades the ER is Atg40/FAM134B [34,35,36]. During ER stress, the canonical branches of the UPR regulate autophagy in different ways [20]. IRE1 cause autophagy by increasing JNK1 [37]. In our study, DOX caused an increase in the expression of JNK1. In addition, Rg1 can inhibit the of JNK1. Because the autophagy-related gene Beclin1 is the leading downstream regulator of JNK1, its expression was also detected [38]. We found that Rg1 can significantly reduce the increase of Beclin1 caused by DOX. Transcription activity of ATF6 is involved in autophagy induction through downregulation of AKT [39]. In the article by Zhu et al., oral administration of Rg1 significantly increased the phosphorylation of AKT in the hearts of mice treated with doxorubicin [18]. In addition, both IRE1 and ATF6 can activate autophagy by affecting the mTOR pathway [20]. In the detection of P70S6K, we found that DOX significantly increased its phosphorylation level. However, the content of p-P70S6K decreased significantly in Rg1 treatment mice hearts when compared with that of DOX. These results suggest that the inhibition of autophagy by Rg1 may be related to its role in endoplasmic reticulum stress. We further examined the changes of FAM134B, the key protein in the ER-phagy [40,41,42]. There was no significant change of FAM134B in the mice hearts in each group. Our results thus hint that ginsenoside Rg1 may inhibit the ER stress-mediated autophagy in DOX-treated mice heart. As mentioned previously, the relationship between autophagy and DOX-induced cardiac dysfunction is complex. In addition, there are many other factors that can cause autophagy. Whether ginsenoside Rg1 can affect autophagy through other ways needs further study.

Although our experiments found that Rg1 inhibited endoplasmic reticulum stress and autophagy in DOX-treated mice hearts, other ways may also be involved in the cardioprotective effect of Rg1. Mitochondrial dysfunction often occurs in heart failure [24,43]. In addition, the improvement of mitochondrial dysfunction by pharmacological methods can ameliorate cardiac dysfunction induced by DOX [44,45]. Ginsenoside Rg1 has been reported to improve mitochondrial dysfunction associated with heart disease. Therefore, the role of ginsenoside Rg1 in regulating mitochondrial function may also be responsible for its improvement of DOX-induced cardiac dysfunction.

## 4. Materials and Methods 

### 4.1. Reagents

Ginsenoside Rg1 (purity of 98%) was purchased from manster biotechnology co., Ltd. (Chengdu, China). Doxorubicin (a purity of 98%) was procured from Aladdin Industrial Company (Shanghai, China). Antibodies against LC3A/B (4108), P70S6K (2708), p-P70S6K (9234), P62 (23214) and Beclin1 (3495) were purchased from Cell Signaling Technology (Shanghai, China). Antibody against ATF6 (sc-22799), XBP1 (sc-7160) and GFAT1 (sc-134894) were purchased from Santa Cruz Biotechnology (Shanghai, China). Antibodies against IRE1 (ab37073), ATG5 (ab108327), TIF1 (ab174287), GRP78 (ab108615), HRD1 (ab170901), FAM134B (ab151755), JNK1 (ab179461) and GAPDH (abb181602) were purchased from Abcam (Shanghai, China).

### 4.2. Animals and Experimental Protocols

All experiments and animal care in this study were conducted in accordance with the Provision and General Recommendation of Chinese Experimental Animals Administration Legislation and approved by the Science and Technology Department of Jiangsu Province (SYXK (SU) 2016-0011). Eight-week-old male wild-type C57BL/6J mice (20–22 g body weight) were obtained from Beijing Vital River Laboratory Animal Technology Co., Ltd. (Beijing, China). The mice were exposed to a 12 h light–12 h dark cycle at 22 ± 2 °C with relative humidity of 60% ± 5%. Food and water were supplied ad libitum. The mice were acclimatized for one week before the experiments.

Thirty mice were randomly divided into 3 groups (*n* = 10 each) as follows: the control group (C), doxorubicin group (DOX), and doxorubicin + Rg1 treatment group (Rg1). The mice in the DOX and DOX + Rg1 groups were injected intraperitoneally with 6 mg/kg of DOX every 3 days, four times total; the control mice received equal volume PBS. Rg1 (50 mg/kg/day, i.g.) was administered 7 days before the first DOX injection and continued until the termination of the study. A schedule of the experiments can be seen in Figure 1B.

### 4.3. Echocardiography

Cardiac function was analyzed under isoflurane anesthesia using the Vevo 2100 high-resolution in vivo imaging system (Visual Sonics, Toronto, ON, Canada) at day 5 after the final injection of DOX or saline. Left ventricular ejection fraction (LVEF) and left ventricular fractional shortening (LVFS) were measured as previously described [45]. After echocardiography, hearts were collected for further study.

### 4.4. Histopathology

After being washed with PBS, the hearts from the mice were fixed were fixed in 10% neutral-buffered formalin at room temperature for 24 h. Then heart tissues were embedded in paraffin, cut into 5 μm sections and stained with hematoxylin and eosin (H&E) to analyze the heart morphology. To evaluate the degree of myocardial fibrosis, heart samples were also stained with Masson′s trichrome. Sections were viewed and imaged with a Nikon Ts2R research microscope.

### 4.5. Electron Microscopy

Transmission electron microscopy was used to examine the autophagy and endoplasmic reticulum. Conventional electron microscopy (EM) technique was used as previously described [46].

### 4.6. RNA Extraction and qPCR

Total RNA was isolated from mouse heart tissue using Trizol reagent (Vazyme, Nanjing, China) according to the manufacturer’s instructions. Two micrograms of total RNA from each sample was reverse transcribed into cDNA using the HiscriptTMQRT Super-Mix for qPCR kit (Vazyme). The resulting cDNAs were amplified using a SYBR Green Master Mix Kit in LightCycler^®^ 96 system (Roche, Switzerland). Primers used for pre-rRNA and β-actin are as follows: Pre-rRNA forward 5′-CTCTTAGATCGATGTGGTGCTC-3′ and reverse 5′-GCCCGCTGGCAGAACGAGAAG-3′, β-actin, forward 5′- GGCACCACACCTTCTACAATG-3′ and reverse 5′-T GGGGTGTTGAAGGTCTCAAC -3′. Gene expression levels were calculated using the 2^−ΔΔCt^ method.

### 4.7. Western Blot

Total proteins were obtained from cardiac tissues as described [47]. Thirty micrograms of total protein were separated by SDS/PAGE and transferred to PVDF membrane. The membrane was then blocked with 5% bovine serum albumin for 2 h at room temperature and incubated at 4 °C overnight with primary antibodies. After three washings with odyssey blocking buffer, the membranes were incubated with secondary fluorescence anti-body (Odyssey, Lincoln, NE, USA) in TBS solution for 60 min at room temperature, then washed as above. After washing, immune reactive protein bands were detected by Odyssey SA (Li-cor, Lincoln, NE, USA) and quantified by Odyssey Image Studio 5.1.

### 4.8. Statistical Analysis

Values are indicated as mean ± SEM. Statistical analysis of multiple groups was performed with one-way ANOVA. All of the statistical tests were performed with the GraphPad Prism software version 7.0 (GraphPad Software, Inc., San Diego, CA, USA). *p* values < 0.05 were considered statistically significant.

## 5. Conclusions

**Figure 7:**
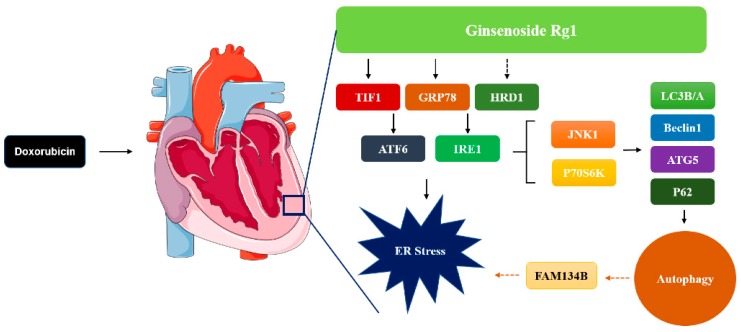
Schematic diagram of how ginsenoside Rg1 attenuates doxorubicin-induced cardiomyotoxicity through the inhibition of autophagy and endoplasmic reticulum stress in mice.

In summary, the present work demonstrates that Rg1 administration can alleviate DOX-induced heart failure (Figure 7). The potential mechanism of its protective role is mediated by inhibition of excessive autophagy and ER stress. Our results indicate that Rg1 may attenuate DOX-induced ER stress by affecting the protein expressions of TIF1 and GRP78. In addition, the role of Rg1 in inhibiting autophagy may be related to its regulation of JNK1 and P70S6k. This suggests Rg1 as a potential agent to treat the cardiac damage of DOX.
